# Visualization of Complex Processes in Cardiovascular System during Electrical Auricular Vagus Nerve Stimulation

**DOI:** 10.3390/diagnostics11122190

**Published:** 2021-11-25

**Authors:** Vaiva Šiaučiūnaitė, Minvydas Ragulskis, Alfonsas Vainoras, Babak Dabiri, Eugenijus Kaniusas

**Affiliations:** 1 Department of Mathematical Modelling, Kaunas University of Technology, 51368 Kaunas, Lithuania; 2 Cardiology Institute, Lithuanian University of Health Sciences, 44307 Kaunas, Lithuania; alfavain@gmail.com; 3 Institute of Electrodynamics, Microwave and Circuit Engineering, Vienna University of Technology, 1040 Vienna, Austria; babak.dabiri.razlighi@tuwien.ac.at (B.D.); eugenijus.kaniusas@tuwien.ac.at (E.K.)

**Keywords:** auricular vagus nerve stimulation, electrocardiography, cardiac intervals

## Abstract

The analysis of human physiological systems from the point of view of complex systems theory remains a very ambitious task. The complexity of the problem often encourages the use of innovative mathematical methods analyzing the processes that take place in space and time. The main goal of this paper is to visualize the cardiovascular system during auricular vagus nerve stimulation (aVNS) using the matrix differences to evaluate the dynamic signal interfaces by cointegrating the initial signal data into the matrices during each case. Algebraic relationships between RR/JT and JT/QRS cardiac intervals are used not only to track the cardiovascular changes during aVNS but also to characterize individual features of the person during the transit through the therapy. This paper presents the computational techniques that can visualize the complex dynamical processes taking place in the cardiovascular system using the electrical aVNS therapy. Four healthy volunteers participated in two verum and two placebo experiments. We discovered that the body’s reaction to the stimulation was very different in each of the cases, but the presented techniques opened new possibilities for a novel interpretation of the dynamics of the cardiovascular system.

## 1. Introduction

The vagus nerve (VN) is the longest cranial nerve in the human body and it is involved in the regulation of multiple systems [1]. Historically, the vagus nerve has been studied as an efferent nerve and as an antagonist of the sympathetic nervous system. Most organs receive parasympathetic afferents through the vagus nerve and sympathetic afferents through the splanchnic nerves. Together with the sympathetic nervous systems, the parasympathetic nervous system is responsible for the regulation of vegetative functions by acting in opposition to each other [2]. Due to this wide influence on multiple systems and its important role in maintaining homeostasis, stimulating the VN to modulate the function of related organs has long drawn the attention of investigators [3].

**Figure 1:**
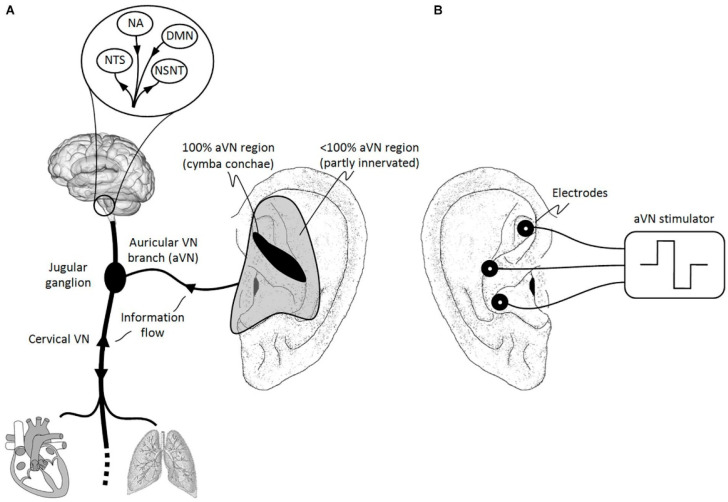
Natural sensory innervation of the auricle versus its artificial stimulation. (**A**) The vagus nerve (VN) connects the brain with most of the organs within the thorax and abdomen. Afferent auricular branches (aVN) leave the cervical VN at the level of the jugular ganglion just outside the cranium and innervate the rather central regions of the pinna of the outer ear (Peuker and Filler, 2002). (**B**) Electric stimulation of aVN endings with needle electrodes located within these central regions. NTS, nucleus of the solitary tract; NSNT, nucleus spinalis of the trigeminal nerve; NA, nucleus ambiguous; DMN, dorsal motor nucleus. This figure and figure caption were originally published in [6], which was published in Frontiers of Neuroscience under the creative commons attribution license CC BY 4.0.

The external ear is the only place on the body where VN sends its only peripheral branch. In fact, the auricular branch of VN surfaces as the afferent auricular VN (aVN) and, thus, forms a cutaneous receptive field in the pinna of the ear. This field is susceptible to external stimuli in terms of peripheral nerve stimulation. In particular, the aVN allows for an easy external access via electrical stimulation in terms of aVNS, which then connects, directly and favorably, the applied stimuli to the brainstem, as shown in Figure 1B. The brainstem even mediates aVNS input to higher brain regions via extensive projections to second and third order neurons within the brain [4]. The auricle, and especially its aVN endings, might become a powerful direct gateway to modulate various brain functions, offering the most affordable non-invasive manipulation of the central nervous system. As shown in Figure 1A, the middle region of the pinna, the central concha, is mostly innervated by the aVN, whereas the aVN was found in 100% of cases in the cymba concha [5].

The human functional state changes as a result of vagus nerve stimulation. In terms of neuromodulation, it recruits sensory aVN fibers and so simulates/projects sensory information to the brainstem, forming the auriculo-vagal afferent pathway [7]. Both the autonomic nervous system (ANS) and the central nervous system (CNS) are influenced by aVNS because it projects directly to the nucleus of the solitary tract (NTS). Since the autonomic nervous system (ANS) is made up of sympathetic and parasympathetic branches, regulating systemic parameters of cardiovascular, respiratory, and immunological functions to keep them within their homeostatic limits, and since aVNS modulates the parasympathetic auricular branch, the effects of aVNS on the body can be expected to be systemic. The VN, which is part of the parasympathetic nervous system, counteracts the sympathetic stress response [8].

The parasympathetic system is activated in several ways, one of which is arterial baroreflex activation. The principle of negative feedback governs the regulation of vascular tone, as it does many other systems. Arterial baroreceptors in the aortic arch transmit blood pressure to the brainstem via the afferent VN, and so provide this as feedback for controlling arterial blood pressure in the near term (baroreflex). The arterial blood pressure (ABP) and vascular tonus often fluctuate in a wave-like pattern due to these causes. Hypertension stimulates baroreceptors in the aortic arch, which send signals to the brainstem through the afferent VN. In reaction to hypertension, the brain, particularly the NTS, restricts sympathetic outflow to the heart and periphery instinctively, whilst stroke volume and total peripheral resistance decrease, respectively. The parasympathetic outflow is automatically enhanced by efferent VN fibers, decreasing the heartbeat through the efferent VN link to the sinoatrial node. As a result, blood pressure falls and returns to normal. Because of these brain-mediated effects, auricular VNS can be used as a systemic therapy, affecting the entire human body and its complex subsystems [9].

However, the individual tuning of stimulation parameters remains an open problem in the practical implementation of aVNS. Several studies have shown that the outcome of therapeutic effects of aVNS is observed in long-term treatment [10,11]. The optimization of the stimulation protocol and parameters remains an open topic in tuning the individualized approach towards the patient’s state. The achievement of optimal treatment outcomes for individual patients, the minimization of effects caused by over-stimulation and under-stimulation could be attained only if the systemic changes in the human body could be observed in real time during the stimulation process [12].

Numerical algorithms for the identification of algebraic relationships between cardiac intervals have already been designed and implemented in several previous studies [13,14,15]. These algorithms are utilized in this paper for the construction of phase plots representing complex dynamical processes during the self-organization of the cardiovascular system. The main objective of this paper is to present a computational framework able to visualize complex reactions of the cardiovascular system during aVNS. The paper is organized as follows: the role and the relationships between cardiac intervals are discussed in the Preliminaries section, the experimental setup, the stimulation protocol, and computational algorithms are presented in the Materials and Methods section. Results and insights for further studies are discussed in the final section.

## 2. Preliminaries

Changes in the patterns of interconnection (connectivity) and patterns of variation over time (variability) offer essential information about the state of the overall system since the spatial and temporal organization of a complex system defines its own character [16]. Measuring the absolute value of a clinical parameter such as the heart rate provides highly important and therapeutically useful data. However, analyzing the interconnections of ECG characteristics provides additional clinical information that is often more informative than heart rate alone, especially when the heart rate is within normal limits [17].

Systemic monitoring methods are required for the registration, validation, and optimization of systemic effects induced by the auricular VNS. Methods of interest that reflect the non-linear complex systems of the human body are coupled mutually with feedback loops [18]. Here, the ECG is of a particular importance. Relationships between ECG parameters do vary according to various physiological and pathological reasons [19].

A single ECG feature, such as the RR interval, which is a systemic parameter, indicates the functional status of the entire organism’s regulatory system and is used as a measure of neurocardiac function that reflects heart–brain interactions and ANS dynamics [20,21]. For example, as the heart fails, not only do the individual regulating systems attenuate (decrease in multiplicity) but also their tight coupling, such as arterial or cardiopulmonary baroreceptor activity, deteriorates, which makes the regulating system and the RR interval less complex [22,23]. Within an organism, an appropriate amount of RR interval fluctuations indicates healthy function as well as innate self-regulatory capacity, flexibility, or resilience [21,24].

In particular, the JT interval as an ECG feature is linked to the metabolic rate in myocardium; namely, when heart activity is the strongest, the JT interval is the shortest and vice versa. The JT interval describes the duration of ventricular repolarization. The JT range determines different electrophysiological phenomena and is divided into the JTa interval (from the point J to the peak of T wave) and the Te interval (from the T wave peak to the end of T). JT intervals are linked to variations in cardiac metabolism [25]. The JT interval is related to the intensity of myocardial metabolism and correlates to cardiac electric systole. The alterations of DJT (duration of JT interval) are influenced by the regulatory nervous system. Metabolic changes in the organism are closely associated with repolarization changes. ECG leads, where the JT interval is shorter, show that in those myocardium areas, the repolarization happens earlier, and metabolic changes are faster. Longer DJT shows slower repolarization and slower metabolic reactions [26]. 

Another ECG parameter, QRS duration, is related to interventricular synchronization features, and could be connected to the heart intrinsic regulatory system. During sympathetic activation, during load in a healthy heart, we can see shortening of this parameter. This prolongs some heart pathologies–ischemic heart disease. The QRS complex is a part of the regulatory system of the heart, which reflects a spreading of the depolarization in the ventricle and synchronization of the depolarization spreading between ventricles. The wider QRS complex shows a slower conduction in the heart ventricle [27]. The duration of the QRS complex can range in normal conditions from 80 to 120 ms. This index is sensitive to sympathetic and parasympathetic nervous system tonus alterations. 

For example, RR–JT interconnection shows how tied the regulatory system is connected to the metabolism of the heart; this relationship reveals the evolution of complex dynamical processes in the self-organization of the heart system during the load and the recovery processes [13]. Additionally, this relationship is used for the detection of prolonged repolarization in ventricular conduction defects [28], but the JT interval is constructed as a linear function of the RR interval in the mentioned study. Similarly, it was shown in [29] that a heart rate’s corrected JT interval was a good estimate of specific repolarization time in a cohort of physically fit university students. There is a well-known parabolic relationship between the HR and JT interval: when the HR rises, the JT interval shortens, and vice versa. This JT interval shortening shows the accelerating metabolism of the heart.

RR–QRS interconnection reflects the interconnection between the organism regulatory system and the heart regulatory system by the reaction of the duration of the QRS complex to the RR interval shortening. It must be kept in mind that certain feedback responses (based on the type of stress induced) may include possible changes in vascular tone, with or without influencing HR, and certain elements of the individual cardiopulmonary and arterial reflexes may involve counteracting or inhibiting influences with regards to other elements [28]. For example, the increased beat-to-beat duration of QRS complex variability has been suggested as a possible marker for myocardial ischemia and infarction [30]. It is believed that the subtle variations may reflect islets of ischemic tissue, variations in sympathetic tone, coronary flow, or myocardial contraction pattern [31]. Connective tissue among myocardial fibers can also possibly influence the electrical activation path from beat to beat.

**Figure 2:**
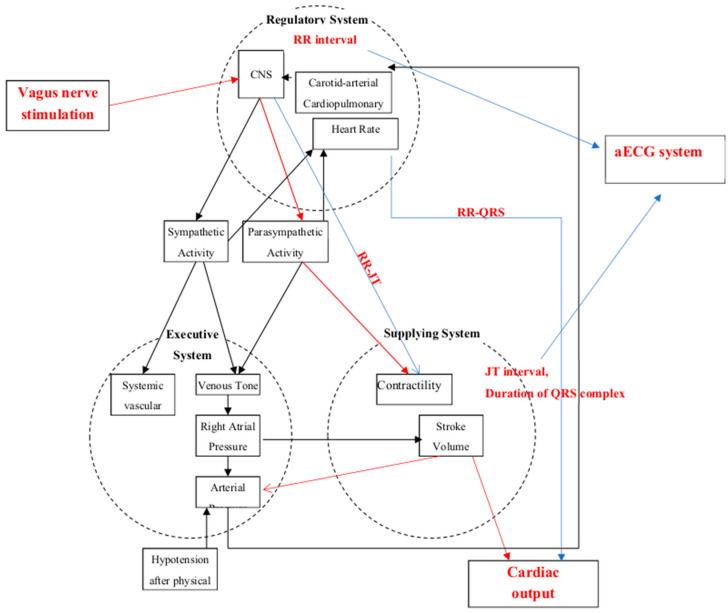
Baroreceptor control loops in the context of the model of integral evaluation.

For the evaluation of the human functional state during vagus nerve stimulation, we used the model of integral health evaluation. This model is shown together with the short-term neural baroreflex feedback loop response model in Figure 2, inspired by [28].

## 3. Materials and Methods

### 3.1. Experimental Setup

The presented research met all standards for the ethics of experimentation. The permit to perform biomedical investigation was granted by Kaunas Regional Ethics Committee for Biomedical Investigations, No. BE-2-51, 23 December 2015. Our study consists of 4 single cases. Each of the volunteers signed a consent form to take part in this research. They were assessed four times, two verum and two placebo experiments. (Table 1). In this study we investigated four healthy persons (two males and two females) at the average age of 27.8 (±2.22).

**Figure 3:**
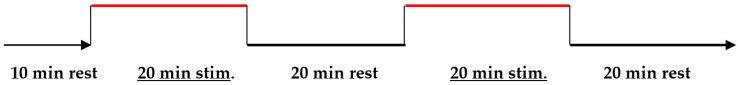
The time protocol of the experiment.

Twelve lead ECG were assessed and analyzed using the Kaunas–Load system [16]. Twenty-five different parameters and their interconnections were measured per cardiac cycle using advanced ECG analysis. The experiment started with a baseline, represented with black line in (Figure 3) recording phase of 10 min without pVNS, continued with a verum phase of 20 min with pVNS (red line), a resting phase of 20 min without pVNS (black line), another verum phase of 20 min (red line), and a follow-up period of recovery phase for 20 min (black line). In total, more than 5000 cardio cycles were recorded and estimated.

**Figure 4:**
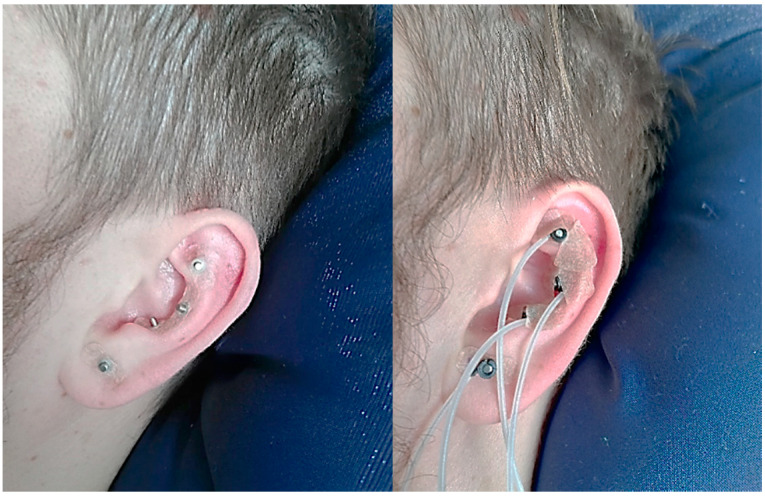
The location of four needle electrodes.

Percutaneous auricular VNS (pVNS) was performed with three needle electrodes located in vagally innervated regions of the auricle, in/around the cymba conchae (Figure 4). Stimulation sequence comprised monophasic pulses of alternating polarity, repeated every 1 s (amplitude 4 V, pulse duration 1 ms).

### 3.2. The Description of the Algorithm

Let us consider that vectors $x=\left(x_{1},x_{2},\ldots ,x_{n}\right)$ and $y=\left(y_{1},y_{2},\ldots ,y_{n}\right)$ do represent synchronous measurement data representing two different cardiac intervals; where n is the total number of heart beats recorded during the experiment. The algebraic relationship between time series x and y is reconstructed using the algorithm presented in [13]. This algorithm comprises three basic parts.

Step #1. Six elements $x_{k-\delta }$, $x_{k}$, $x_{k+\delta }$, $y_{k-\delta }$, $y_{k}$, $y_{k+\delta }$ are mapped into a two-dimensional perfect matrix of Lagrange differences (the concept of perfect matrices of Lagrange differences is introduced in [13], where δ is the time lag; k=(1+δ),(2+δ),…,(n−δ). The structure of the perfect matrix of Lagrange differences is [13]

(1) $$L_{\delta ,k}=\left[\begin{array}{cc} x_{k} & x_{k+\delta }-y_{k+\delta }\\ x_{k-\delta }-y_{k-\delta } & y_{k} \end{array}\right]$$

Step #2. Every matrix $L_{\delta ,k}$ is transformed into a scalar value $s_{\delta ,k}=max\left(\left|\lambda_{1}\right|,\left|\lambda_{2}\right|\right)$ where $\lambda_{1}$ and $\lambda_{2}$ are the two eigenvalues of $L_{\delta ,k}$ [13,14]. 

Step #3. Finally, internal and external smoothing is applied for the produced scalar sequence of maximum eigenvalues. If the radius of the internal smoothing is denoted by $R_{i}$ and the radius of the external smoothing is denoted by $R_{e}$, then the smoothed sequence depicting the algebraic relationship between time series x and y reads

(2) $$\bar{s_{k}}\left(R_{i},R_{e}\right)=\frac{1}{R_{i}\left(2R_{e}+1\right)}\overset{k+R_{e}}{\underset{j=k-R_{e}}{\sum }}\overset{R_{i}}{\underset{\delta =1}{\sum }}s_{\delta ,j}$$

where $k=\left(1+R_{i}+R_{e}\right),\left(2+R_{i}+R_{e}\right),\ldots ,\left(n-R_{i}-R_{e}\right)$. A well-posed optimization problem in respect of the smoothing parameters is formulated in [13] for the whole cohort of persons resulting in $R_{i}=3$ and $R_{e}=4$. These values of the smoothing parameters are kept fixed in this paper too. All further analysis is based on $\bar{s_{k}}\left(3,4\right)$.

## 4. Results

Case reports suggest that the right vagus can be used in circumstances where approaching the left vagus is inadvisable. Since the right vagus innervates the sinoatrial node, stimulating on the right is best conducted with ECG monitoring [32]. It is well known that phase maps of bioelectrical signals may be useful for the investigation of dynamical processes [33]. In order to better represent the attractors (and to assess their stability) in this paper we also visualize the dynamical processes of vagus nerve stimulation in a phase plane. The graphs below show the full dynamics of the vagus nerve stimulation experiment.

**Figure 5:**
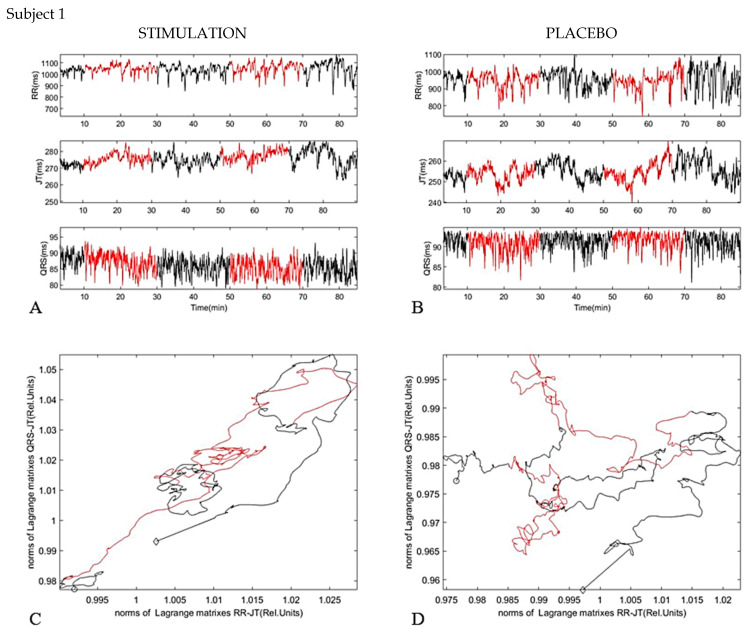
Subject 1. (**A**) dynamic of parameters RR interval, JT interval, and QRS duration during investigation (Stimulation). (**B**) dynamic of parameters RR interval, JT interval, and QRS duration during investigation (Placebo). (**C**) phase plane for JT/QRS and RR/JT norms during stimulation. (**D**) phase plane for JT/QRS and RR/JT norms during placebo. The beginning of the experiment is marked as a black circle, the end of the experiment marked as a black diamond marker. In total, more than 5000 cardio cycles were recorded and estimated.

In the first case, we can observe that when the body relaxes, the curve goes to the upper right corner (Figure 5). Both JT–QRS and RR–JT relations values are increasing (Figure 5). After the first stimulation, the black curve (rest after first stimulation) makes a loop (Figure 5), circuiting in one place, then the red curve, the second stimulation phase, goes up again. This means the independency of the two systems, and the complexity of the parameters increases, then, during the recovery again, it makes a loop to go back to the initial body phase state. A different behavior we see when we compare the stimulation session with the placebo session. There are no clear changes in the process dynamics, but there are small fluctuations about initial body state.

**Figure 6:**
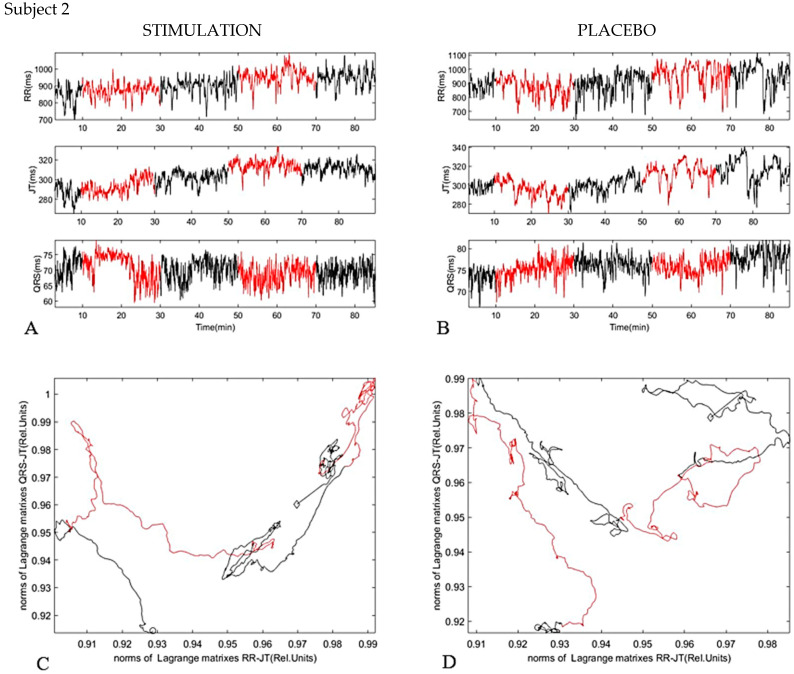
Subject 2. (**A**) dynamic of parameters RR interval, JT interval, and QRS duration during investigation (Stimulation). (**B**) dynamic of parameters RR interval, JT interval, and QRS duration during investigation (Placebo). (**C**) phase plane for JT/QRS and RR/JT norms during stimulation. (**D**) phase plane for JT/QRS and RR/JT norms during placebo.

The second subject shows more complicated changes: during the second stimulation, the tension in both fractal levels (RR/JT and JT/QRS) is also reduced (Figure 6). The system in the phase plane goes to the right-top part of Figure 6. During the sham stimulation (Placebo), the black curves make a loop, but the general trend for both systems is to go into a state of relaxation. If it was just a lying effect, the black line at the end of the experiment would not go back, it always rises (effect of long-lasting rest).

**Figure 7:**
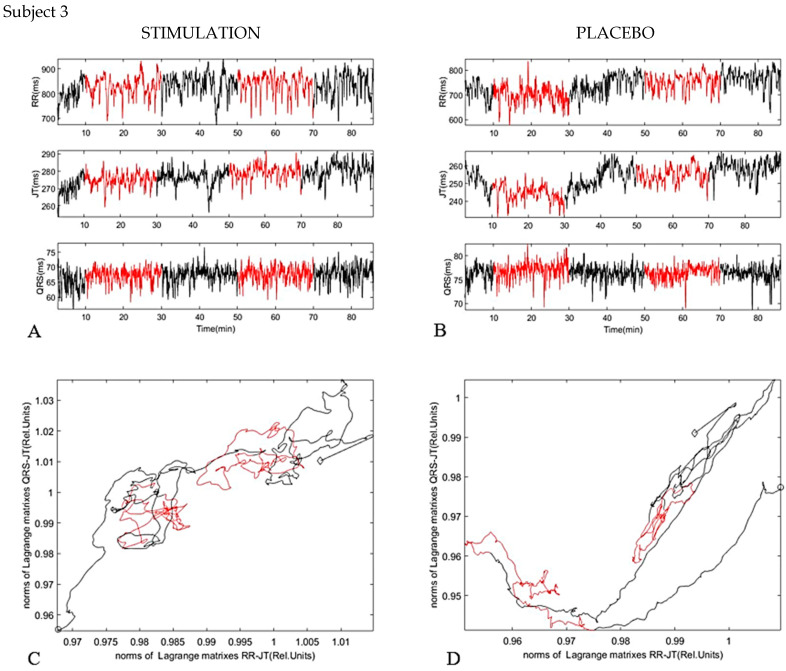
Subject 3. (**A**) dynamic of parameters RR interval, JT interval, and QRS duration during investigation (Stimulation). (**B**) dynamic of parameters RR interval, JT interval, and QRS duration during investigation (Placebo). (**C**) phase plane for JT/QRS and RR/JT norms during stimulation. (**D**) phase plane for JT/QRS and RR/JT norms during placebo.

The third case (Figure 7) has a classical pattern of the vagus nerve stimulation dynamics that appeared in most of the cases. The complexity between relations grew, the systems became more independent from each other. In the sham stimulation (placebo) we saw a similar effect as in the real stimulation. This could be due to the insertion of the needles providing excitation to the parasympathetic nervous system; the body already has a memory of the effect of the needle’s penetration.

**Figure 8:**
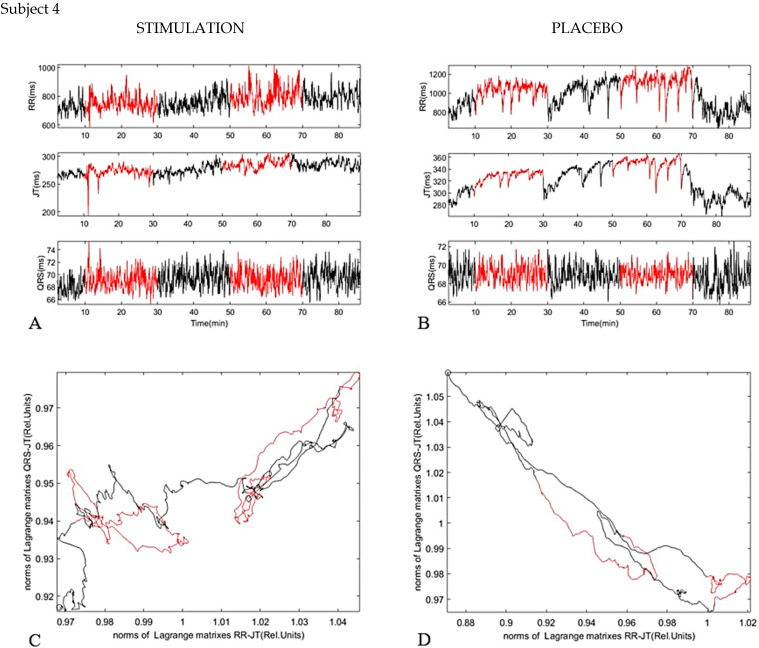
Subject 4. (**A**) dynamic of parameters RR interval, JT interval, and QRS duration during investigation (Stimulation). (**B**) dynamic of parameters RR interval, JT interval, and QRS duration during investigation (Placebo). (**C**) phase plane for JT/QRS and RR/JT norms during stimulation. (**D**) phase plane for JT/QRS and RR/JT norms during placebo.

The fourth subject showed the classical behavior in the same way as the first stimulation, however, for some reason the second stimulation did not show much effect (second red curve) (Figure 8). This could be due to many factors, for example psychological state or stressful thoughts during the experiment.

## 5. Discussion

Complex systems have properties that cannot fully be comprehended by understanding the parts of the system [25]. The properties of the system are distinct from the properties of the parts, and they depend on the integrity of the whole; the systemic properties vanish when the system breaks apart, whereas the properties of the parts are maintained. Every fractal level subsystem has its own local regulatory mechanisms. Subsystems integrate the activity of the vasomotor and respiratory autonomous oscillators, with reflex neural commands occurring in response to changes in some controlled variables (e.g., arterial pressure) and with humoral factors. ECG parameters can be categorized into systemic, indicating changes at the highest fractal level of the body, and peripheral, indicating changes at the level of the organs or related to them [18]. Estimating the absolute value of a clinical parameter such as heart rate gives extremely important and therapeutically relevant information. However, analyzing the interconnections of the ECG features provides additional clinical information that is often more relevant than the heart rate alone, especially when the heart rate is within normal ranges [12,17]. It is well known that phase maps of bioelectrical signals may be useful for the investigation of dynamical processes [31]. In order to better represent the attractors (and to assess their stability) in this paper, we also visualize the dynamical processes of vagus nerve stimulation in a phase plane. The graphs above showed the full dynamics of the vagus nerve stimulation experiment.

When it is possible to objectively measure the body’s reactions, the parameters such as at the time of stimulation can be optimized. Even though the human body’s reaction to any stimulus could be very different and unique, with our visualization technique we could successfully track the whole process of the electrical auricular vagus nerve stimulation in two different fractal levels of the human organism. 

### Strengths and Limitations

The proposed methodology for monitoring subtle variations of algebraic relationships between cardiac intervals during aVNS is able to track individual reactions of the patient in real time. The presented techniques provide a unique insight into the complex dynamical processes taking place in a patient’s cardiovascular system during the stimulation. The ability to track these dynamical processes can build the ground for the optimization strategy, which could lead to personalized aVNS treatment. The development of such a personalized approach to aVNS treatment is a definite objective of future research.

The main limitation of this paper was the size of the cohort. As mentioned previously, the main objective of this study is to present the methodology for tracking physiological changes during aVNS in real time. Cardiac intervals play the major role in the presented technique. In other words, the proposed technique does not measure the direct impact of the nerve stimulation to targeted systems under the planned treatment. Instead, the proposed technique visualizes the real-time subtle reactions of the cardiovascular system. However, the cardiovascular system is affected not only by aVNS during the stimulation. The patient is asked to stay calm in a supine position during the whole experiment. The fatigue, drowsiness, excitement, and the momentary pain caused by the insertion of the needle electrodes are all factors that have an integrated impact on the cardiovascular system. Therefore, the observed dynamic relationships represent the integrated response to all external and internal stimuli. In other words, the presented technique cannot produce reliable results if the patient is not in a calm state. Further studies, including extensive clinical trials are required before validated optimization algorithms can be designed for personalized aVNS treatment.

The process of using only the ECG signal, which does not require any additional measurements, is non-invasive, revealing information in a short-term measurement.

Unfortunately, we did not have a large cohort to investigate. The psychological state of the subject fluctuated because the experiment required a supine position for 90 min.

## Figures and Tables

**Table 1:** The description of subjects participating in the experiment.

Gender	Age, y	Height, m	Weight, kg	BMI, kg/m^2^
M	31	1.81	83	25.34
F	27	1.72	65	21.97
M	26	1.77	65	20.75
F	27	1.69	55	19.26

## Data Availability

All datasets analyzed in the current study are available from the corresponding author on reasonable request.
